# Impact of rapid Cepheid Xpert Xpress severe acute respiratory coronavirus virus 2 (SARS-CoV-2) reverse-transcription polymerase chain reaction (RT-PCR) testing in Detroit Medical Center hospitals during the coronavirus disease 2019 (COVID-19) pandemic

**DOI:** 10.1017/ash.2022.291

**Published:** 2022-09-13

**Authors:** Monica P. Meyer, Jennifer LeRose, Catherine Maples, Teena Chopra

**Affiliations:** 1 Wayne State University School of Medicine, Detroit, Michigan; 2 Michigan State University, College of Osteopathic Medicine, East Lansing, Michigan; 3 Division of Infectious Disease, Department of Internal Medicine, Detroit Medical Center, Detroit, Michigan

## Abstract

In March 2020, the coronavirus disease 2019 (COVID-19) pandemic raged, and samples from the Detroit Medical Center (DMC) were sent offsite for testing. From April 3, 2020, DMC laboratories ran rapid Cepheid Xpert Xpress severe acute respiratory coronavirus virus 2 (SARS-CoV-2) reverse-transcription polymerase chain reaction (RT-PCR) tests within hospital labs. We detected differences in length of stay (LOS) and antibiotic duration between positive results from offsite and in-house tests.

Numerous cases of pneumonia of unknown origin were detected in China in December 2019.^1^ These cases were quickly linked to the novel virus, now known as severe acute respiratory syndrome coronavirus 2 (SARS-CoV-2). Coronavirus disease 2019 (COVID-19) cases rapidly spread worldwide and officially became a global pandemic in March 2020. To date, >89 million cases of COVID-19 in the United States have been confirmed as well as >1 million associated deaths.^2^

Detroit, Michigan, was especially affected by the pandemic, ranking third in the country for confirmed cases during March and April 2020.^3^ The first case of COVID-19 in Michigan was detected at the Detroit Medical Center (DMC) on March 11, 2020. Initially, nasopharyngeal swab samples were sent to outside laboratories, and the turnaround time was ∼7–10 days. As the DMC became flooded with cases through March and into early April, this offsite testing turnaround time became increasingly untenable.

As COVID-19 cases continued to increase in Detroit, timely and accurate SARS-CoV-2 laboratory testing became an essential part of the management of COVID-19 because it helped to identify and contain viral spread. Supporting decisions on infection control strategies, patient management at healthcare facilities, and detecting asymptomatic cases that could cause further spread if not isolated are also benefits of timely testing. The rapid Cepheid Xpert Xpress SARS-CoV-2 reverse-transcription polymerase chain reaction (RT-PCR) test (Cepheid, Sunnyvale, CA) was implemented and run in house at the DMC on April 3, 2020. This test provided a faster turnaround time than offsite testing. In this study, we compared hospital length of stay (LOS) and duration of antibiotics between SARS-CoV-2–positive COVID-19 patients who underwent testing via Cepheid Xpert Xpress PCR performed at DMC and those who did not (ie, samples were sent to external laboratories).

## Methods

We performed a descriptive study using the DMC COVID-19 patient registry. The patient registry was developed by the Wayne State University Organized Research Consortium to Study COVID-19 (WORCS-C) through medical record extraction by trained healthcare staff. Patients included in the registry were all SARS-CoV-2–positive COVID-19 cases identified at a DMC institution between March 11, 2020, and May 14, 2020. Institutional review board approval was obtained.

### Length of stay

The distribution of LOS was compared between groups as hours, days, and log transformed LOS, as well as with emergency department (ED) and inpatient stratifications. Generalized linear mixed models were used to test for differences in LOS with adjustment for patient-level characteristics. Post hoc analyses modeled median differences with quantile regression to account for skewness. Results were not appreciably different and thus are not reported.

### Antibiotic duration

General linear models were fit to determine whether the mean number of days on antibiotic treatment differed among patients who tested positive for SARS-CoV-2 using either a Cepheid test or an alternative.

## Results

Among the 1,473 COVID-19 patients included in the study, 925 (63%) received offsite SARS-CoV-2 testing (March 11–April 3, 2020) and 548 (37%) received Cepheid Xpert Xpress SARS-CoV-2 PCR testing (April 3–May 14, 2020). The offsite testing group was 53% male; 70% were aged 60 years and older; 59% were African American, and 33% were obese (Table 1).

**Table 1.** Descriptive Characteristics of Patient Encounters by Test Type

Characteristic	Offsite Test (n = 925)		Cepheid Rapid PCR (n = 548)		Overall (n = 1,473)	
	No.	%	No.	%	No.	%
**Sex**						
Female	452	48.9	257	46.9	709	48.1
Male	473	51.1	290	52.9	763	51.8
Unknown	0		1	0.2	1	0.1
**Age groups**						
0–4 y	5	0.5	6	1.1	11	0.7
5–17 y	4	0.4	4	0.7	8	0.5
18–24 y	5	0.5	6	1.1	11	0.7
25–39 y	67	7.2	37	6.8	104	7.1
40–49 y	102	11.0	36	6.6	138	9.4
50–59 y	155	16.8	77	14.1	232	15.8
60–69 y	250	27.0	144	26.3	394	26.7
70–79 y	196	21.2	135	24.6	331	22.5
≥80 y	141	15.2	103	18.8	244	16.6
**Race**						
Black	562	60.8	325	59.3	887	60.2
Other/Unknown	322	34.8	179	32.7	501	34.0
White	41	4.4	44	8.0	85	5.8
**BMI**						
Class I obesity	162	17.5	81	14.8	243	16.5
Class II obesity	115	12.4	52	9.5	167	11.3
Class III obesity	141	15.2	48	8.8	189	12.8
Not documented	111	12.0	87	15.9	198	13.4
Overweight	219	23.7	123	22.4	342	23.2
Underweight	21	2.3	25	4.6	46	3.1
Z-normal weight	156	16.9	132	24.1	288	19.6
**Smoking status**						
Nonsmoker	844	91.2	492	89.8	1,336	90.7
Regular smoker	81	8.8	56	10.2	137	9.3

Note. PCR, polymerase chain reaction assay; BMI, body mass index.

Without adjustment for patient-level characteristics, the LOS was significantly greater in the Cepheid-test study group than among patients whose nasopharyngeal swabs were tested by external laboratories: LOS median, 6.9 days (interquartile range [IQR], 2–14) versus 5.3 days (IQR, 2–10) and least squares mean log 2 (LOS), 2.7 versus 2.5 days (*P* = .01). The LOS remained significantly elevated among patients who were tested using the Cepheid test after adjusting for age and race categories, the total number of clinical diagnoses (an indicator of case-mix severity) and obesity: least squares mean log 2 (LOS), 2.4 versus 2.2 days (*P* = .02).

**Fig. 1.**
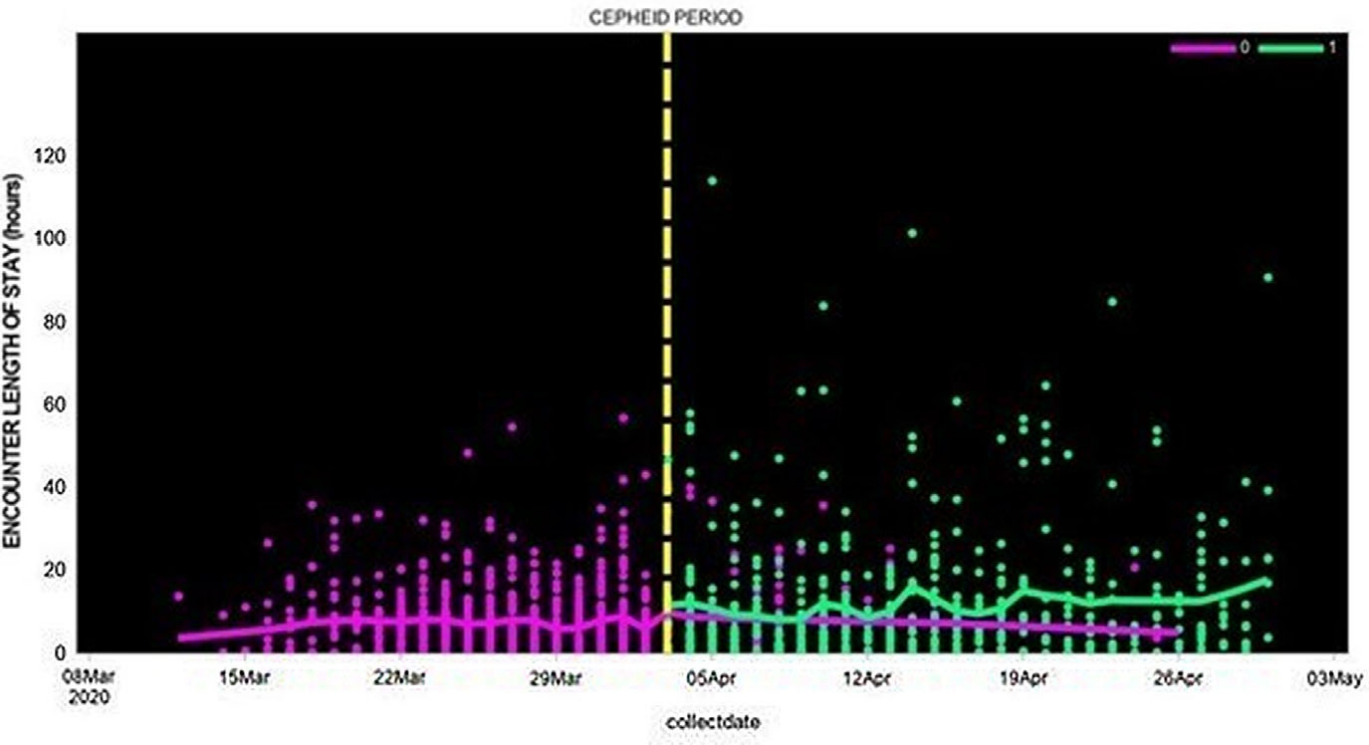
Length of hospital stay among patients whose SAR-CoV-2 test was performed using Cepheid rapid PCR or by external laboratories (March 12, 2020, to April 30, 2020).

In contrast to total LOS, the emergency department (ED) LOS was lower in the Cepheid-test study group compared to patients whose nasal swabs were tested by external laboratories: the ED LOS median was 26 (IQR, 4–21) versus 36 hours (IQR, 2–10), the least-squares mean log 2 (LOS) was 0.7 versus 1.0 days (*P* = .01), and the adjusted mean log 2 (LOS) was 0.4 versus 0.7 days (*P* < .0001) (Fig. 1).

Overall, the average number of days on antibiotic treatment differed significantly between patients who did and did not test positive for SARS-CoV-2 by the Cepheid test depending on their age group (interaction *P* = .003) and adjusting for potentially confounding factors (ie, race category and the sum of clinical diagnoses).

Children (aged <18 years) who were identified by the Cepheid test received antibiotics for 3 fewer days than their same-age peers who were not identified by the Cepheid test (*P* = .01). By contrast, patients aged 50–64 years received antibiotics for 2.7 more days than their peers of the same age who were detected by an alternative test (*P* = .03). We did not detect differences in the duration of antibiotics treatment by test type among patients aged 18–49 years (difference, 1 day; *P* = .41) or ≥65 years (difference, 0.9 day; *P* = .06).

## Discussion

Our study has demonstrated multiple benefits that correlate with shorter test-turnaround time provided by the Cepheid Xpert Xpress SARS-CoV-2 rapid PCR that was run in house. First, the data show shorter LOS for ED patients within the in-house cohort. We suspect that readily available SARS-CoV-2 laboratory results allowed clinicians to create more accurate treatment plans and to swiftly discharge patients who were confirmed SARS-CoV-2 negative by the Cepheid test. The Cepheid test results also likely allowed for quicker implementation of isolation precautions for patients that tested positive, helping to curb the spread of the virus.

Interestingly, the LOS for admitted COVID-19 patients was greater within the Cepheid test cohort. We suspect that this seemingly counterintuitive finding can be explained as follows. (1) Better treatment options for COVID-19 treatment became available as the pandemic progressed, such as dexamethasone in severely ill patients, increasing survival which in turn prolonged LOS. (2) Public awareness surrounding COVID-19 infection increased; thus, patients likely sought medical treatment earlier in their disease course. And (3) positive SARS-CoV-2 PCR results may have expedited admission orders for COVID-19 patients.^4^

Additionally, Cepheid testing was correlated with shorter duration of antibiotic therapy for select patient populations. Pneumonia caused by SARS-CoV-2 virus mimics bacterial pneumonia; both have constitutional and respiratory symptoms. However, antibiotics are not indicated for the treatment of COVID-19 pneumonia.^4^ Therefore, we suspect that clinicians initiated antibiotics for presumptive bacterial pneumonia upon patient arrival and stopped antibiotic therapy upon notification of positive SARS-CoV-2 PCR results. Consequently, the faster turnaround time provided by the Cepheid testing allowed for reduced antibiotic administration.

Our study had several limitations. First, DMC is an urban teaching hospital with a unique patient population; thus, our results may not be generalizable to rural hospitals. Additionally, we did not examine potentially confounding factors related to the pandemic, such as personal protective equipment shortages and changes in triage procedures, due to the inability to retroactively quantify these variables. Nevertheless, we were able to demonstrate statistically significant differences between cohorts regarding LOS and antibiotic usage.

These data provide preliminary evidence to support the use of Cepheid Xpert Xpress SARS-CoV-2 PCR testing in hospitals. The benefits include decreased ED LOS and antibiotic usage. Both factors are essential in minimizing resource exhaustion and containing the continued spread of the SARS-CoV-2 virus. Further research with larger studies that address confounding factors is needed to understand this correlation.
